# Supplementary material for: Dietary Constituents: Relationship with Breast Cancer Prognostic (MCC-SPAIN Follow-Up)
Source: Int J Environ Res Public Health. 2020 Dec 24;18(1):84. doi: 10.3390/ijerph18010084 (PMC7794807; doi:10.3390/ijerph18010084)
Supplement: Supplementary file 1 [file ijerph-18-00084-s001.pdf]

Table S1. Tumour characteristics.

Variable	Category	N (%)
**Intrinsic subtype**	Luminal A	853(63.19)
	Luminal B	260(19.26)
	Her2-non luminal	67(4.96)
	Basal-like	103(7.63)
	Luminal ONI	45(3.33)
	Non-luminal ONI	5(0.37)
	Missing	17(1.26)
**Pathological Prognosis Stage**	IA	503(37.26)
	IB	124(9.19)
	IIA	60(4.44)
	IIB	39(2.89)
	IIIA	32(2.37)
	IIIB	17(1.26)
	IIIC	4(0.30)
	IV	28(2.07)
	Non applicable (Neoadjuvancy)	121(8.96)
	Missing	422(31.26)
**Histology**	Ductal	1090(80.74)
	Lobular	91(6.74)
	Paget disease	14(1.04)
	Others	155(11.48)
**Grade**	I: well differentiated	293(21.70)
	II: moderately differentiated	422(31.26)
	III: poorly differentiated	277(20.52)
	Missing	358(26.52)
**Estrogen Receptor**	Negative	192(14.22)
	Positive	1140(84.44)
	Missing	18(1.33)
**Progesterone Receptor**	Negative	311(23.04)
	Positive	1018(75.41)
	Missing	21(1.56)
**Her 2**	Negative	1060(78.52)
	Positive	226(16.74)
	Missing	64(4.74)

ONI: Otherwise non-identified. Luminal ONI: hormonal receptors positive, Her2 missing. Non-luminal ONI: hormonal receptors negative, Her2 missing.

Table S2. Daily nutrient intake in the study population

Nutrient	All women	Survivors women	Death women
Carbohydrates intake (g/day), mean (SD)	192.63 (2.11)	191.76(2.26)	198.61(5.90)
Polysaccharides intake (g/day), mean (SD)	86.94 (1.16)	86.88(1.24)	87.36(3.23)
Monosaccharides intake (g/day), mean(SD)	109.18 (1.3)	108.50(1.39)	113.80(3.63)
Proteins intake (g/day), mean(SD)	80.21(0.67)	80.33(0.72)	79.44(1.88)
Animal proteins intake (g/day), mean(SD)	54.33 (0.51)	54.48(0.55)	53.29(1.43)
Vegetable proteins intake (g/day), mean(SD)	25.9 (0.3)	25.86(0.32)	26.16(0.83)
Fats intake (g/day), mean(SD)	83.19 (0.96)	83.27(1.03)	82.62(2.69)
Monounsaturated fats intake (g/day), mean(SD)	37.97 (0.47)	37.94(0.51)	38.22(1.32)

Polyunsaturated fats intake (g/day), mean(SD)	13.97 (0.21)	13.98(0.22)	13.90(0.57)
Saturated fat intake (g/day), mean(SD)	24.34 (0.31)	24.46(0.33)	23.54(0.85)
Energy intake (kcal/day), mean (SD)	1862.51 (14.83)	1861.10(15.87)	1872.21(41.67)
Ethanol intake (g/day), mean (SD) current	4.62 (0.23)	4.71(0.25)	4.02(0.65)

Table S3. Baseline characteristics by tertile (T) of energy intake

		Tertil of energy (Kcal/day)			
**Variable**	**Category**	**T1**	**T2**	**T3**	**p-value**
Geographical area, n (%)	Madrid	100(22.22)	82(18.22)	85(18.89)	.006
	Barcelona	58(12.89)	82(18.22)	80(17.78)	
	Navarra	43(9.56)	59(13.11)	60(13.33)	
	Guipuzkoa	74(16.44)	65(14.44)	52(11.56)	
	Leon	65(14.44)	67(14.89)	73(16.22)	
	Asturias	28(6.22)	26(5.78)	10(2.22)	
	Huelva	15(3.33)	11(2.44)	22(4.89)	
	Cantabria	32(7.11)	40(8.89)	32(7.11)	
	Valencia	19(4.22)	9(2.00)	22(4.89)	
	Girona	16(3.56)	9(2.00)	14(3.11)	
Educational level, n (%)	No schooling	88(19.56)	55(12.22)	52(11.56)	<.001
	Primary school	165(36.67)	138(30.67)	149(33.11)	
	Secondary school	123(27.33)	160(35.56)	162(36.00)	
	University	74(16.44)	97(21.56)	87(19.33)	
Socio-economic status	High	58(12.89)	74(16.44)	72(16.00)	.018
	Middle	223(49.56)	250(55.56)	247(54.89)	
	Low	169(37.56)	126(28.00)	131(29.11)	
Tobacco smoking, n (%)	Never smoked	252(57.27)	254(57.08)	229(51.81)	.359
	Former smoker	101(22.95)	110(24.72)	124(28.05)	
	Current smoker	87(19.77)	81(18.20)	89(20.14)	
Menopausal status	Premenopausal	137(30.44)	167(37.11)	183(40.76)	0.005
	Posmenopausal	313 (69.56)	283 (62.89)	266(59.24)	
Family history of breast cancer, n (%)	None	362(80.80)	308(68.90)	335(74.44)	<.001
	First-degree relative	52(11.61)	82(18.34)	58(12.89)	
	Second-degree relative	34(7.59)	57(12.75)	57(12.67)	
Previous use of hormonal contraceptives, n (%)	Never	220(55.14)	218(53.83)	195(48.03)	.099
	Ever	179(44.86)	187(46.17)	211(51.97)	
Hormone Replacement Therapy in postmenopausal women	Never	353(88.47)	368(90.86)	374(92.12)	.398
	Ever	32(8.02)	28(6.91)	25(6.16)	
	Not Known (or not remember)	14(3.51)	9(2.22)	7(1.72)	
Age, mean (SD)		57.93(0.59)	55.49(0.59)	54.08(0.59)	<.001
Body Mass Index (kg/m2), mean (SD)		26.48(0.23)	25.71(0.23)	25.52(0.23)	0.01
Energy intake (kcal/day), mean (SD)		1332.75(13.12)	1783.22(13.12)	2471.55(13.12)	<.001
Carbohydrates intake (g/day)		139.46(2.08)	181.43(2.08)	249.96(2.08)	<.001
Polysaccharides intake (g/day))		62.59(19.62)	81.00(20.58)	113.11(41.11)	<.001
Monosaccharides intake (g/day)		80.18(22.74)	104.16(26.92)	139.85(43.64)	<.001
Proteins intake (g/day)		62.18(0.71)	77.58(0.71)	98.87(0.71)	<.001
Animal proteins intake (g/day)		42.72(11.70)	52.87(12.98)	66.37(16.66)	<.001
Vegetable proteins intake (g/day)		19.49(5.19)	24.75(6.07)	32.50(8.84)	<.001
Fats intake (g/day)		54.98(0.91)	77.78(0.91)	114.03(0.91)	<.001
Monounsaturated fats intake (g/day)		24.77(9.11)	36.11(9.82)	52.10(15.01)	<.001
Polyunsaturated fats intake (g/day)		9.19(3.27)	12.78(3.74)	19.30(7.40)	<.001
Saturated fat intake (g/day)		16.12(4.44)	22.37(4.79)	33.53(8.99)	<.001

Ethanol intake (g/day), mean (SD) current	3.23(0.41)	5.18(0.41)	5.53(0.41)	<.001

Table S4. Influence of carbohydrate intake on breast cancer survival according to menopausal status, BMI and oestrogen receptor status. Hazard ratios comparing third and second tertiles vs. first tertile of consumption

Sample	Type of carbohydrate		1st tertile	HR (95% CI) 2nd tertile	HR (95% CI) 3rd tertile	p trend
Premenopausal	**Total carbohydrates**	Deaths/woman-years	16/920	13/1087	17/1223	
		Model 1	1(ref.)	0.92 (0.37 to 2.29)	1.07 (0.31 to 3.67)	0.93
		Model 2	1(ref.)	0.95(0.38 to 2.41	1.12 (0.32 to 3.98)	0.88
		Model 3*	1(ref.)	0.98 (0.35 to 2.71)	1.29 (0.28 to 6.00)	0.78
	**Monosaccharides**	Deaths/woman-years	15/1113	18/958	13/1158	
		Model 1	1(ref.)	1.63 (0.73 to 3.62)	0.78 (0.25 to 2.42)	0.86
		Model 2	1(ref.)	1.76 (0.80 to 3.90)	0.68 (0.21 to 1.87)	0.80
		Model 3#	1(ref.)	1.64 (0.74 to 3.65)	0.61 (0.19 to 1.97)	0.65
	**Polysaccharides**	Deaths/woman-years	15/854	15/1030	16/1345	
		Model 1	1(ref.)	0.70 (0.29 to 1.66)	0.56 (0.20 to 1.56)	0.27
		Model 2	1(ref.)	0.73 (0.29 to 1.82)	0.64 (0.22 to 1.87)	0.42
		Model 3$	1(ref.)	0.67 (0.26 to 1.77)	0.59 (0.19 to 1.79)	0.37
Postmenopausal	**Total carbohydrates**	Deaths/woman-years	36/2013	40/1859	49/1683	
		Model 1	1(ref.)	1.25 (0.75 to 2.08)	1.38 (0.75 to 2.56)	0.30
		Model 2	1(ref.)	1.25 (0.75 to 2.08)	1.32 (0.70 to 2.48)	0.38
		Model 3*	1(ref.)	1.28 (0.74 to 2.21)	1.38 (0.63 to 3.01)	0.40
	**Monosaccharides**	Deaths/woman-years	41/1797	37/1984	47/1774	
		Model 1	1(ref.)	0.70 (0.43 to 1.15)	0.72 (0.41 to 1.25)	0.25
		Model 2	1(ref.)	0.68 (0.41 to 1.12)	0.72 (0.41 to 1.27)	0.27
		Model 3#	1(ref.)	0.69 (0.42 to 1.15)	0.70 (0.39 to 1.26)	0.24
	**Polysaccharides**	Deaths/woman-years	39/2130	50/1840.5	36/1584	
		Model 1	1(ref.)	1.52 (0.97 to 2.41)	1.06 (0.60 to 1.89)	0.73
		Model 2	1(ref.)	1.50 (0.94 to 2.37)	1.08 (0.60 to 1.92)	0.70
		Model 3$	1(ref.)	1.57 (0.98 to 2.50)	1.04 (0.58 to 1.88)	0.77
BMI≤25	**Total carbohydrates**	Deaths/woman-years	16/1219	23/1422	23/1429	
		Model 1	1(ref.)	1.65 (0.78 to 3.49)	3.96 (1.45 to 10.9)	<0.01
		Model 2	1(ref.)	1.47 (0.69 to 3.13)	3.68 (1.33 to 10.2)	0.02
		Model 3*	1(ref.)	1.59 (0.69 to 3.65)	3.36 (1.01 to 11.2)	0.05
	**Monosaccharides**	Deaths/woman-years	16/1366	24/1293	22/1412	
		Model 1	1(ref.)	1.94 (0.97 to 3.88)	1.95 (0.85 to 4.46)	0.10
		Model 2	1(ref.)	1.95 (0.95 to 4.00)	2.12 (0.88 to 5.09)	0.08
		Model 3#	1(ref.)	2.22 (1.04 to 4.72)	2.59 (1.04 to 6.48)	0.04
	**Polysaccharides**	Deaths/woman-years	20/1247	26/1315	16/1508	
		Model 1	1(ref.)	1.18 (0.61 to 2.29)	0.74 (0.31 to 1.75)	0.54
		Model 2	1(ref.)	1.02 (0.52 to 1.99)	0.69 (0.29 to 1.65)	0.44
		Model 3$	1(ref.)	1.03 (0.51 to 2.08)	0.74 (0.30 to 1.86)	0.56
BMI>25	**Total carbohydrates**	Deaths/woman-years	29/1473	26/1310	39/1314	
		Model 1	1(ref.)	0.71 (0.38 to 1.30)	0.89 (0.44 to 1.82)	0.77
		Model 2	1(ref.)	0.66 (0.35 to 1.24)	0.89 (0.43 to 1.85)	0.74
		Model 3*	1(ref.)	0.76 (0.39 to 1.49)	1.19 (0.48 to 2.95)	0.79
	**Monosaccharides**	Deaths/woman-years	31/1301	30/1529	33/1268	
		Model 1	1(ref.)	0.50 (0.29 to 0.89)	0.45 (0.23 to 0.91)	0.03
		Model 2	1(ref.)	0.53 (0.29 to 0.95)	0.50 (0.24 to 1.02)	0.06
		Model 3#	1(ref.)	0.60 (0.33 to 1.10)	0.57 (0.27 to 1.19)	0.14
	**Polysaccharides**	Deaths/woman-years	30/1552	32/1309	32/1236	
		Model 1	1(ref.)	1.01 (0.58 to 1.77)	1.10 (0.57 to 2.09)	0.78
		Model 2	1(ref.)	0.99 (0.55 to 1.76)	1.12 (0.58 to 2.17)	0.74
		Model 3$	1(ref.)	0.97 (0.54 to 1.75)	1.03 (0.52 to 2.04)	0.94
ER negative	**Total carbohydrates**	Deaths/woman-years	17/316	11/415	21/400	
		Model 1	1(ref.)	0.55 (0.21 to 1.39)	1.02 (0.32 to 3.24)	1.00
		Model 2	1(ref.)	0.54 (0.19 to 1.50)	1.26 (0.34 to 4.65)	0.83
		Model 3*	1(ref.)	0.41 (0.12 to 1.37)	1.28 (0.26 to 6.34)	0.76
	**Monosaccharides**	Deaths/woman-years	17/340	15/328	17/463	
		Model 1	1(ref.)	0.94 (0.40 to 2.20)	0.47 (0.17 to 1.34)	0.17
		Model 2	1(ref.)	0.70 (0.29 to 1.71)	0.40 (0.13 to 1.18)	0.10
		Model 3#	1(ref.)	0.79 (0.32 to 1.99)	0.40 (0.13 to 1.20)	0.11
	**Polysaccharides**	Deaths/woman-years	15/367	19/347	15/417	
		Model 1	1(ref.)	1.19 (0.47 to 3.01)	0.79 (0.28 to 2.19)	0.59
		Model 2	1(ref.)	1.76 (0.62 to 5.00)	0.84 (0.27 to 2.65)	0.71

		Model 3$	1(ref.)	2.12 (0.67 to 6.70)	0.84 (0.25 to 2.81)	0.63
ER positive	**Total carbohydrates**	Deaths/woman-years	35/2571	42/2490	44/2486	
		Model 1	1(ref.)	1.41 (0.84 to 2.35)	1.52 (0.78 to 2.97)	0.21
		Model 2	1(ref.)	1.38 (0.83 to 2.30)	1.43 (0.73 to 2.81)	0.29
		Model 3*	1(ref.)	1.49 (0.86 to 2.56)	1.83 (0.83 to 4.08)	0.12
	**Monosaccharides**	Deaths/woman-years	39/2520	39/2571	43/2457	
		Model 1	1(ref.)	0.84 (0.52 to 1.35)	0.94 (0.52 to 1.68)	0.82
		Model 2	1(ref.)	0.83 (0.51 to 1.37)	0.93 (0.51 to 1.68)	0.79
		Model 3#	1(ref.)	0.86 (0.52 to 1.42)	0.92 (0.50 to 1.70)	0.77
	**Polysaccharides**	Deaths/woman-years	39/2567	46/2502	36/2479	
		Model 1	1(ref.)	1.20 (0.76 to 1.90)	0.85 (0.47 to 1.55)	0.66
		Model 2	1(ref.)	1.21 (0.76 to 1.92)	0.88 (0.49 to 1.60)	0.74
		Model 3$	1(ref.)	1.19 (0.74 to 1.92)	0.85 (0.46 to 1.55)	0.63

Model 1: Adjusted for hospital of recruitment, age, PPS score (0, IA, IB,IIA, IIB, IIA, IIB, IIC,IV,non applicable, missing), systemic treatment received by the patients: chemotherapy (yes, non), hormone therapy (yes, non) and immunology therapy (yes, non) and total energy intake (Kcal/day) one year before the diagnosis **Model 2:** Adjusted for all previous variables and socioeconomic status (low, middle, high), education attained, physical activity (metabolic equivalents (METs)) during the 5 years before diagnosis, smoking status one year before recruitment (never; former; current) and Body Mass Index(kg/m^2). **Model 3:** Adjusted for the same variables in model 2 and diabetes (yes, non, unknown), and *percentage of calories from the other major nutrient groups, # percentage of calories from polysaccharides, $ percentage of calories from monosaccharides.

Table S5. Influence of protein intake on breast cancer survival according to menopausal status, BMI and oestrogen receptor status. Hazard ratios comparing third and second tertiles vs. first tertile of consumption

Sample	Type of protein		1st tertile	HR (95% CI) 2nd tertile	HR (95% CI) 3rd tertile	p trend
Premenopausal	**Total proteins**	Deaths/woman-years	18/888	14/1116	14/1225	
		Model 1	1(ref.)	0.70 (0.31 to 1.60)	0.63 (0.21 to 1.92)	0.39
		Model 2	1(ref.)	0.65 (0.27 to 1.59)	0.61 (0.19 to 1.97)	0.39
		Model 3*	1(ref.)	0.60 (0.24 to 1.48)	0.47 (0.13 to 1.73)	0.23
	**Animal proteins**	Deaths/woman-years	16/870	17/1090	13/1269	
		Model 1	1(ref.)	0.66 (0.29 to 1.50)	0.66 (0.27 to 1.65)	0.38
		Model 2	1(ref.)	0.65 (0.28 to 1.52)	0.66 (0.25 to 1.74)	0.41
		Model 3#	1(ref.)	0.51 (0.21 to 1.25)	0.47 (0.17 to 1.32)	0.16
	**Vegetable proteins**	Deaths/woman-years	21/985	11/1134	12/1110	
		Model 1	1(ref.)	0.43 (0.18 to 1.02)	0.66 (0.23 to 1.91)	0.29
		Model 2	1(ref.)	0.40 (0.16 to 0.97)	0.57 (0.19 to 1.67)	0.21
		Model 3$	1(ref.)	0.35 (0.14 to 0.86)	0.47 (0.15 to 1.43)	0.12
Postmenopausal	**Total proteins**	Deaths/woman-years	42/2000	38/1840	45/1715	
		Model 1	1(ref.)	1.08 (0.66 to 1.75)	1.31 (0.71 to 2.40)	0.41
		Model 2	1(ref.)	1.06 (0.65 to 1.74)	1.30 (0.70 to 2.41)	0.42
		Model 3*	1(ref.)	1.18 (0.70 to 1.98)	1.62 (0.79 to 3.35)	0.20
	**Animal proteins**	Deaths/woman-years	36/2058	47/1812	42/1686	
		Model 1	1(ref.)	1.85 (1.16 to 2.96)	1.53 (0.88 to 2.67)	0.10
		Model 2	1(ref.)	1.88 (1.17 to 3.03)	1.47 (0.84 to 2.58)	0.15
		Model 3#	1(ref.)	1.88 (1.17 to 3.04)	1.47 (0.82 to 2.63)	0.16
	**Vegetable proteins**	Deaths/woman-years	41/1909	41/1824	43/1822	
		Model 1	1(ref.)	1.20 (0.75 to 1.92)	0.80 (0.45 to 1.43)	0.52
		Model 2	1(ref.)	1.24 (0.77 to 1.98)	0.84 (0.46 to 1.51)	0.65
		Model 3$	1(ref.)	1.25 (0.78 to 2.01)	0.86 (0.47 to 1.57)	0.72
BMI≤25	**Total proteins**	Deaths/woman-years	22/1283	25/1441	15/1347	
		Model 1	1(ref.)	1.31 (0.67 to 2.56)	0.99 (0.37 to 2.64)	0.89
		Model 2	1(ref.)	1.60 (0.79 to 3.25)	1.20 (0.43 to 3.37)	0.59
		Model 3*	1(ref.)	1.73 (0.79 to 3.76)	1.39 (0.41 to 4.63)	0.49
	**Animal proteins**	Deaths/woman-years	23/1396	26/1353	13/1322	
		Model 1	1(ref.)	1.37 (0.73 to 2.58)	0.69 (0.28 to 1.70)	0.60
		Model 2	1(ref.)	1.64 (0.84 to 3.20)	0.79 (0.31 to 1.98)	0.81

		Model 3#	1(ref.)	1.68 (0.85 to 3.35)	0.82 (0.32 to 2.10)	0.85
	**Vegetable proteins**	Deaths/woman-years	22/1224	21/1463	19/1384	
		Model 1	1(ref.)	0.76 (0.38 to 1.53)	1.08 (0.46 to 2.55)	0.93
		Model 2	1(ref.)	0.68 (0.33 to 1.41)	1.05 (0.44 to 2.50)	0.98
		Model 3$	1(ref.)	0.66 (0.32 to 1.37)	0.99 (0.41 to 2.41)	0.93
BMI>25	**Total proteins**	Deaths/woman-years	31/1378	24/1318	39/1401	
		Model 1	1(ref.)	0.69 (0.38 to 1.26)	0.96 (0.48 to 1.91)	0.83
		Model 2	1(ref.)	0.74 (0.40 to 1.37)	1.01 (0.50 to 2.06)	0.96
		Model 3*	1(ref.)	0.75 (0.40 to 1.43)	1.09 (0.49 to 2.41)	0.90
	**Animal proteins**	Deaths/woman-years	24/1334	33/1332	37/1431	
		Model 1	1(ref.)	1.41 (0.79 to 2.52)	1.41 (0.74 to 2.69)	0.31
		Model 2	1(ref.)	1.52 (0.84 to 2.78)	1.53 (0.79 to 2.95)	0.23
		Model 3#	1(ref.)	1.54 (0.85 to 2.82)	1.41 (0.72 to 2.76)	0.34
	**Vegetable proteins**	Deaths/woman-years	34/1415	25/1333	35/1349	
		Model 1	1(ref.)	0.81 (0.47 to 1.40)	0.56 (0.29 to 1.08)	0.09
		Model 2	1(ref.)	0.76 (0.43 to 1.33)	0.61 (0.31 to 1.21)	0.15
		Model 3$	1(ref.)	0.76 (0.43 to 1.35)	0.63 (0.32 to 1.25)	0.18
ER negative	**Total proteins**	Deaths/woman-years	17/1312	15/394	17/425	
		Model 1	1(ref.)	0.87 (0.39 to 1.94)	0.56 (0.18 to 1.74)	0.34
		Model 2	1(ref.)	1.09 (0.44 to 2.67)	0.77 (0.22 to 2.62)	0.75
		Model 3*	1(ref.)	1.59 (0.58 to 4.38)	1.48 (0.33 to 6.66)	0.54
	**Animal proteins**	Deaths/woman-years	14/276	23/448	12/407	
		Model 1	1(ref.)	1.26 (0.57 to 2.80)	0.34 (0.10 to 1.13)	0.19
		Model 2	1(ref.)	1.91 (0.79 to 4.63)	0.27 (0.07 to 1.06)	0.31
		Model 3#	1(ref.)	1.74 (0.71 to 4.26)	0.24 (0.06 to 0.99)	0.23
	**Vegetable proteins**	Deaths/woman-years	18/363	14/367	17/401	
		Model 1	1(ref.)	1.05 (0.46 to 2.40)	0.71 (0.26 to 1.93)	0.56
		Model 2	1(ref.)	1.13 (0.44 to 2.91)	0.80 (0.26 to 2.45)	0.77
		Model 3$	1(ref.)	0.94 (0.34 to 2.61)	0.77 (0.26 to 2.33)	0.66
ER positive	**Total proteins**	Deaths/woman-years	43/2540	37/2526	41/2483	
		Model 1	1(ref.)	0.96 (0.58 to 1.58)	1.15 (0.61 to 2.19)	0.69
		Model 2	1(ref.)	0.90 (0.54 to 1.50)	1.11 (0.58 to 2.14)	0.77
		Model 3*	1(ref.)	0.95 (0.55 to 1.63)	1.31 (0.62 to 2.76)	0.52
	**Animal proteins**	Deaths/woman-years	38/2618	41/2434	42/2495	
		Model 1	1(ref.)	1.30 (0.80 to 2.09)	1.48 (0.86 to 2.56)	0.16
		Model 2	1(ref.)	1.31 (0.80 to 2.14)	1.45 (0.84 to 2.52)	0.19
		Model 3#	1(ref.)	1.30 (0.79 to 2.14)	1.44 (0.82 to 2.54)	0.21
	**Vegetable proteins**	Deaths/woman-years	44/2494	38/2549	39/2505	
		Model 1	1(ref.)	0.92 (0.57 to 1.49)	0.78 (0.42 to 1.43)	0.42
		Model 2	1(ref.)	0.94 (0.58 to 1.53)	0.78 (0.42 to 1.45)	0.44
		Model 3$	1(ref.)	0.95 (0.58 to 1.54)	0.79 (0.42 to 1.49)	0.48

Model 1: Adjusted for hospital of recruitment, age, PPS score (0, IA, IB,IIA, IIB, IIA, IIB, IIC,IV,non applicable, missing), systemic treatment received by the patients: chemotherapy (yes, non), hormone therapy (yes, non) and immunology therapy (yes, non) and total energy intake (Kcal/day) one year before the diagnosis **Model 2:** Adjusted for all previous variables and socioeconomic status (low, middle, high), education attained, physical activity (metabolic equivalents (METs)) during the 5 years before diagnosis, smoking status one year before recruitment (never; former; current) and Body Mass Index (kg/m2). **Model 3**: Adjusted for the same variables in model 2 and *percentage of calories from the other major nutrient groups, # percentage of calories from vegetable protein, $ percentage of calories from animal protein.

Table S6 Influence of fat intake on breast cancer survival according to menopausal status, BMI and oestrogen receptor status. Hazard ratios comparing third and second tertiles vs. first tertile of consumption

Sample	Type of fat		1st tertile	HR (95% CI) 2nd tertile	HR (95% CI) 3rd tertile	p trend

Premenopausal	**Total fats**	Deaths/woman-years	20/985	9/907	17/1338	
		Model 1	1(ref.)	0.54 (0.20 to 1.47)	0.67 (0.19 to 2.32)	0.50
		Model 2	1(ref.)	0.53 (0.19 to 1.53)	0.64 (0.17 to 2.41)	0.50
		Model 3*	1(ref.)	0.25 (0.08 to 0.84)	0.20 (0.04 to 0.98)	0.06
	**Saturated fats**	Deaths/woman-years	13/777	18/1075	15/1377	
		Model 1	1(ref.)	1.51 (0.61 to 3.74)	0.95 (0.25 to 3.57)	0.99
		Model 2	1(ref.)	1.47 (0.57 to 3.819	0.83 (0.21 to 3.19)	0.83
		Model 3#	1(ref.)	1.28 (0.48 to 3.41)	0.65 (0.16 to 2.67)	0.58
	**Monounsaturated fats**	Deaths/woman-years	28/1001	7/1032	19/1196	
		Model 1	1(ref.)	0.44 (0.15 to 1.26)	1.74 (0.57 to 5.35)	0.34
		Model 2	1(ref.)	0.46 (0.15 to 1.39)	1.50 (0.48 to 4.74)	0.48
		Model 3$	1(ref.)	0.45 (0.14 to 1.41)	1.49 (0.46 to 4.82)	0.44
	**Polyunsaturated fats**	Deaths/woman-years	15/824	15/1082	16/1323	
		Model 1	1(ref.)	0.47 (0.20 to 1.09)	0.54 (0.18 to 1.62)	0.25
		Model 2	1(ref.)	0.50 (0.21 to 1.21)	0.60 (0.19 to 1.93)	0.34
		Model 3£	1(ref.)	0.46 (0.19 to 1.12)	0.49(0.14 to 1.66)	0.22
Postmenopausal	**Total fats**	Deaths/woman-years	39/1917	50/2006	36/1631	
		Model 1	1(ref.)	0.92 (0.56 to 1.51)	0.66 (0.30 to 1.45)	0.52
		Model 2	1(ref.)	0.94 (0.57 to 1.53)	0.82 (0.39 to 1.71)	0.60
		Model 3*	1(ref.)	1.00 (0.56 to 1.78)	0.92 (0.37 to 2.29)	0.88
	**Saturated fats**	Deaths/woman-years	50/2132	38/1834	37/1590	
		Model 1	1(ref.)	0.68 (0.42 to 1.09)	0.83 (0.41 to 1.69)	0.41
		Model 2	1(ref.)	0.65 (0.40 to 1.06)	0.78 (0.37 to 1.62)	0.33
		Model 3#	1(ref.)	0.63 (0.38 to 1.04)	0.77 (0.37 to 1.60)	0.30
	**Monounsaturated fats**	Deaths/woman-years	36/1900	57/1894	32/1761	
		Model 1	1(ref.)	1.15 (0.71 to 1.86)	0.62 (0.32 to 1.22)	0.21
		Model 2	1(ref.)	1.20 (0.73 to 1.95)	0.67 (0.33 to 1.33)	0.32
		Model 3$	1(ref.)	1.23 (0.74 to 2.02)	0.69 (0.34 to 1.40)	0.38
	**Polyunsaturated fats**	Deaths/woman-years	15/824	15/1082	16/1323	
		Model 1	1(ref.)	0.97 (0.61 to 1.55)	0.80 (0.43 to 1.48)	0.50
		Model 2	1(ref.)	1.02 (0.63 to 1.64)	0.89 (0.48 to 1.65)	0.72
		Model 3£	1(ref.)	0.98 (0.60 to 1.60)	0.85 (0.45 to 1.61)	0.63
BMI≤25	**Total fats**	Deaths/woman-years	25/1231	21/1307	16/1533	
		Model 1	1(ref.)	0.52 (0.24 to 1.12)	0.32 (0.10 to 1.01)	0.05
		Model 2	1(ref.)	0.58 (0.27 to 1.26)	0.36 (0.11 to 1.17)	0.08
		Model 3*	1(ref.)	0.63 (0.25 to 1.60)	0.43 (0.11 to 1.73)	0.23
	**Saturated fats**	Deaths/woman-years	26/1244	21/1322	15/1505	
		Model 1	1(ref.)	0.68 (0.33 to 1.37)	0.35 (0.11 to 1.11)	0.08
		Model 2	1(ref.)	0.68 (0.33 to 1.40)	0.31 (0.10 to 1.02)	0.06
		Model 3#	1(ref.)	0.77 (0.36 to 1.62)	0.34 (0.10 to 1.13)	0.10
	**Monounsaturated fats**	Deaths/woman-years	23/1244	23/1362	16/1464	
		Model 1	1(ref.)	0.80 (0.39 to 1.65)	0.59 (0.22 to 1.61)	0.30

		Model 2	1(ref.)	0.83 (0.39 to 1.75)	0.62 (0.22 to 1.77)	0.38
		Model 3$	1(ref.)	1.02 (0.46 to 2.26)	0.80 (0.27 to 2.38)	0.69
	**Polyunsaturated fats**	Deaths/woman-years	24/1133	23/1467	15/1471	
		Model 1	1(ref.)	0.56 (0.28 to 1.13)	0.33 (0.13 to 0.85)	0.02
		Model 2	1(ref.)	0.60 (0.30 to 1.21)	0.37 (0.14 to 0.97)	0.04
		Model 3£	1(ref.)	0.64 (0.31 to 1.32)	0.39 (0.15 to 1.02)	0.05
BMI>25	**Total fats**	Deaths/woman-years	31/1447	28/1377	35/1273	
		Model 1	1(ref.)	0.73 (0.41 to 1.30)	0.85 (0.38 to 1.91)	0.60
		Model 2	1(ref.)	0.79 (0.44 to 1.43)	0.98 (0.43 to 2.21)	0.85
		Model 3*	1(ref.)	0.68 (0.35 to 1.32)	0.75 (0.28 to 2.02)	0.50
	**Saturated fats**	Deaths/woman-years	32/1474	28/1331	34/1292	
		Model 1	1(ref.)	0.75 (0.42 to 1.33)	1.05 (0.47 to 2.33)	0.96
		Model 2	1(ref.)	0.78 (0.43 to 1.42)	1.17 (0.51 to 2.67)	0.86
		Model 3#	1(ref.)	0.71 (0.38 to 1.31)	1.07 (0.46 to 2.49)	0.97
	**Monounsaturated fats**	Deaths/woman-years	30/1449	32/1282	32/1366	
		Model 1	1(ref.)	0.91 (0.52 to 1.61)	0.71 (0.35 to 1.47)	0.36
		Model 2	1(ref.)	0.95 (0.54 to 1.69)	0.77 (0.37 to 1.63)	0.52
		Model 3$	1(ref.)	0.94 (0.52 to 1.70)	0.76 (0.35 to 1.64)	0.49
	**Polyunsaturated fats**	Deaths/woman-years	24/1133	23/1467	15/1471	
		Model 1	1(ref.)	0.89 (0.50 to 1.56)	0.77 (0.38 to 1.58)	0.49
		Model 2	1(ref.)	1.01 (0.56 to 1.81)	0.99 (0.47 to 2.07)	0.98
		Model 3£	1(ref.)	0.94 (0.52 to 1.71)	0.91 (0.43 to 1.94)	0.81
ER negative	**Total fats**	Deaths/woman-years	17/325	15/377	17/429	
		Model 1	1(ref.)	0.78 (0.33 to 1.86)	1.20 (0.34 to 4.28)	0.96
		Model 2	1(ref.)	1.31 (0.45 to 3.79)	1.71 (0.37 to 7.87)	0.49
		Model 3*	1(ref.)	1.05 (0.29 to 3.85)	1.14 (0.17 to 7.76)	0.89
	**Saturated fats**	Deaths/woman-years	19/325	13/362	17/444	
		Model 1	1(ref.)	0.66 (0.26 to 1.66)	0.76 (0.21 to 2.75)	0.61
		Model 2	1(ref.)	0.70 (0.26 to 1.87)	0.47 (0.11 to 1.97)	0.30
		Model 3#	1(ref.)	0.39 (0.13 to 1.17)	0.24 (0.05 to 1.15)	0.07
	**Monounsaturated fats**	Deaths/woman-years	16/348	16/329	17/454	
		Model 1	1(ref.)	1.10 (0.47 to 2.56)	1.04 (0.33 to 3.23)	0.92
		Model 2	1(ref.)	2.25 (0.79 to 6.42)	1.53 (0.37 to 6.33)	0.49
		Model 3$	1(ref.)	2.57 (0.83 to 7.98)	1.87 (0.39 to 8.87)	0.41
	**Polyunsaturated fats**	Deaths/woman-years	16/338	15/353	18/441	
		Model 1	1(ref.)	0.67 (0.28 to 1.61)	1.24 (0.41 to 3.75)	0.85
		Model 2	1(ref.)	0.72 (0.28 to 1.89)	1.32 (0.43 to 4.10)	0.74
		Model 3£	1(ref.)	0.57 (0.21 to 1.55)	1.05 (0.32 to 3.50)	0.92
ER positive	**Total fats**	Deaths/woman-years	42/2557	43/2479	36/2512	
		Model 1	1(ref.)	0.86 (0.52 to 1.44)	0.77 (0.36 to 1.66)	0.49
		Model 2	1(ref.)	0.87 (0.52 to 1.47)	0.76 (0.35 to 1.65)	0.49
		Model 3*	1(ref.)	0.81 (0.45 to 1.48)	0.66 (0.26 to 1.70)	0.39

**Saturated fats**	Deaths/woman-years	44/2566	42/2468	35/2515	
	Model 1	1(ref.)	0.91 (0.55 to 1.50)	0.87 (0.41 to 1.84)	0.69
	Model 2	1(ref.)	0.89 (0.54 to 1.48)	0.82 (0.38 to 1.78)	0.60
	Model 3#	1(ref.)	0.85 (0.51 to 1.43)	0.79 (0.36 to 1.72)	0.53
**Monounsaturated fats**	Deaths/woman-years	40/2524	48/2557	33/2466	
	Model 1	1(ref.)	0.97 (0.59 to 1.59)	0.76 (0.39 to 1.46)	0.41
	Model 2	1(ref.)	0.97 (0.58 to 1.61)	0.76 (0.39 to 1.49)	0.42
	Model 3$	1(ref.)	0.96 (0.57 to 1.62)	0.74 (0.37 to 1.47)	0.38
**Polyunsaturated fats**	Deaths/woman-years	16/337.5	15/352.7	18/440.5	
	Model 1	1(ref.)	0.88 (0.55 to 1.43)	0.64 (0.35 to 1.20)	0.17
	Model 2	1(ref.)	0.93 (0.57 to 1.52)	0.72 (0.39 to 1.36)	0.32
	Model 3£	1(ref.)	0.88 (0.54 to 1.46)	0.68 (0.35 to 1.29)	0.24

Model 1: Adjusted for hospital of recruitment, age, PPS score (0, IA, IB,IIA, IIB, IIA, IIB, IIC,IV,non applicable, missing), systemic treatment received by the patients: chemotherapy (yes, non), hormone therapy (yes, non) and immunology therapy (yes, non) and total energy intake (Kcal/day) one year before the diagnosis **Model 2:** Adjusted for all previous variables and socioeconomic status (low, middle, high), education attained, physical activity (metabolic equivalents (METs)) during the 5 years before diagnosis, smoking status one year before recruitment (never; former; current) and Body Mass Index (kg/m^2). **Model 3**: Adjusted for the same variables in model 2 and *percentage of calories from the other major nutrient groups, # percentage of calories from monounsaturated and polyunsaturated fats $ percentage of calories from saturated and polyunsaturated fats **£** percentage of calories from saturated and monounsaturated fats.
